# Federated Learning for IoMT-Enhanced Human Activity Recognition with Hybrid LSTM-GRU Networks

**DOI:** 10.3390/s25030907

**Published:** 2025-02-03

**Authors:** Fahad R. Albogamy

**Affiliations:** Computer Sciences Program, Department of Mathematics, Turabah University College, Taif University, P.O. Box 11099, Taif 21944, Saudi Arabia; f.alhammdani@tu.edu.sa

**Keywords:** federated learning, HAR, human activity recognition, hybrid LSTM-GRU, Internet of Medical Things, IoMT, privacy preservation

## Abstract

The proliferation of wearable sensors and mobile devices has fueled advancements in human activity recognition (HAR), with growing importance placed on both accuracy and privacy preservation. In this paper, the author proposes a federated learning framework for HAR, leveraging a hybrid Long Short-Term Memory (LSTM) and Gated Recurrent Unit (GRU) model to enhance feature extraction and classification in decentralized environments. Utilizing three public datasets—UCI-HAR, HARTH, and HAR7+—which contain diverse sensor data collected from free-living activities, the proposed system is designed to address the inherent privacy risks associated with centralized data processing by deploying Federated Averaging for local model training. To optimize recognition accuracy, the author introduces a dual-feature extraction mechanism, combining convolutional blocks for capturing local patterns and a hybrid LSTM-GRU structure to detect complex temporal dependencies. Furthermore, the author integrates an attention mechanism to focus on significant global relationships within the data. The proposed system is evaluated on the three public datasets—UCI-HAR, HARTH, and HAR7+—achieving superior performance compared to recent works in terms of F1-score and recognition accuracy. The results demonstrate that the proposed approach not only provides high classification accuracy but also ensures privacy preservation, making it a scalable and reliable solution for real-world HAR applications in decentralized and privacy-conscious environments. This work showcases the potential of federated learning in transforming human activity recognition, combining advanced feature extraction methodologies and privacy-respecting frameworks to deliver robust, real-time activity classification.

## 1. Introduction

Human activity recognition (HAR) is becoming increasingly relevant in fields ranging from healthcare to smart environments [1] where the ability to accurately monitor and analyze physical activities can significantly improve personalized services and enhance overall well-being [2]. As wearable devices and sensors continue to proliferate, the potential for sensor-based HAR has grown exponentially, particularly within the context of the Internet of Medical Things (IoMT) [3]. Human activity recognition plays a crucial role in applications such as patient monitoring [4], elderly care [5], and fitness tracking [6] where real-time and accurate activity recognition is vital [7].

However, one of the major challenges in human activity recognition systems lies in balancing high recognition accuracy with privacy concerns as centralized data collection and processing can expose sensitive user information [8]. Traditional machine learning approaches rely on centralizing data from multiple users, which creates risks of privacy breaches and data misuse [9]. To mitigate these risks, federated learning (FL) has emerged as a powerful solution, enabling decentralized model training where user data remain on local devices [10,11]. This ensures that sensitive information is not transmitted to central servers while still allowing collective learning across multiple users [12].

In this paper, the author presents a new framework for human activity recognition, leveraging federated learning combined with advanced feature extraction techniques for real-time analysis. Federated learning enables privacy-preserving, decentralized training, which is crucial for safeguarding sensitive user data in IoMT environments. The system is built upon a hybrid Long Short-Term Memory (LSTM) [13] and Gated Recurrent Unit (GRU) model [14], which excels in capturing the temporal dependencies inherent in sensor data. The hybrid LSTM-GRU model leverages the strengths of LSTM for capturing long-term dependencies and GRU for computational efficiency, making it suitable for processing sequential data in resource-constrained scenarios. By integrating convolutional blocks for local feature extraction and a hybrid LSTM-GRU network for global temporal analysis, the proposed framework is designed to enhance recognition accuracy while preserving user privacy through decentralized learning. This integration also tackles knowledge gaps related to handling imbalanced, heterogeneous, and distributed data while ensuring real-time performance and scalability in decentralized IoMT setups where centralized solutions are either impractical or privacy-invasive.

The proposed federated learning system is evaluated on three public datasets—UCI-HAR, HARTH, and HAR7+ datasets—which represent a rich collection of real-world sensor data capturing diverse human activities in free-living environments. Experimental results demonstrate the superiority of the proposed hybrid model, which significantly outperforms existing federated learning systems in terms of recognition accuracy and privacy preservation. The proposed framework also includes a perceptive extraction network (PEN) for feature extraction [15], ensuring that critical activity patterns are captured effectively across heterogeneous datasets.

The contributions of this work are as follows:

1. Propose a federated learning framework for human activity recognition in IoMT environments using a hybrid LSTM-GRU model, preserving privacy while achieving high classification accuracy.
2. Utilize the PEN approach to enhance feature extraction, combining 1D-CNN for local features and LSTM-based attention for global relationships.
3. Address class imbalance across multiple HAR datasets by applying and evaluating multiple strategies through Weighted Federated Averaging.
4. Demonstrate the adaptability of the framework to diverse datasets (HARTH, HAR70+, UCI-HAR) and highlight the performance improvements in minority class recognition.
5. Provide a comprehensive comparison of class imbalance-handling techniques within the federated learning context, showcasing improvements in accuracy, F1-score, and recall.

By combining federated learning with powerful feature extraction methodologies, the proposed approach paves the way for scalable, privacy-preserving HAR systems that can be deployed in real-world IoMT environments, improving the quality of human activity monitoring while safeguarding user privacy.

The rest of the paper is organized as follows: Section 2 provides an overview of related work in human activity recognition, particularly focusing on privacy-preserving methods and federated learning systems. Section 3 details the proposed federated learning framework for human activity recognition, highlighting the hybrid LSTM-GRU model and the feature extraction techniques employed. Section 4 presents the experimental setup and results from evaluating the proposed model on multiple HAR datasets. In Section 5, the author benchmarked the results with recent and related studies. Section 6 discusses the findings, providing insights into the performance and implications of the federated learning approach, and finally, Section 7 concludes the paper, outlining future research directions and potential applications.

## 2. Related Works

Human activity recognition research leveraging wearable sensors and federated learning has gained considerable attention in recent years due to advancements in IoT technologies and privacy-preserving data analytics. This section reviews significant contributions in HAR systems, federated learning frameworks, and feature extraction techniques pertinent to IoMT-enhanced HAR, contextualizing them within the scope of this work on a hybrid LSTM-GRU model. In selecting the related works for this study, the author strategically focused on research that employed the same public datasets utilized in the proposed framework, specifically HARTH, HAR70+, and UCI-HAR. This selection criterion facilitates direct and meaningful comparisons of methodologies and performance outcomes, thereby underscoring the unique contributions and advancements of the proposed framework. Emphasis was also placed on the most recent studies published mostly between 2023 and 2024, allowing us to incorporate the latest advancements and emerging trends in human activity recognition.

Wavelet transformations are a powerful tool for HAR due to their time–frequency localization, as illustrated by the research work in [16]. The authors emphasize that choosing an optimal mother wavelet function is vital for accurate activity classification, presenting a selection methodology that combines wavelet packet transforms with energy-to-Shannon entropy and using Decision Tree and Support Vector Machine classifiers. Although the wavelet transformation itself is computationally efficient, the overall methodology in [16], which involves wavelet packet decomposition, feature extraction using energy-to-Shannon entropy, and model training with complex classifiers, can become computationally intensive. This makes it less suitable for real-time applications in resource-constrained, decentralized IoT environments. In contrast, this research leverages federated learning combined with neural networks to enhance real-time HAR performance without compromising privacy, offering a more scalable and efficient approach for decentralized systems. Privacy in federated learning is central to the works’ framework.

Chen et al. [17] proposed a novel lightweight machine unlearning technique to enhance federated learning-based human activity recognition models by selectively erasing specific portions of a client’s training data. This approach leverages a separate, third-party dataset that is not involved in the original training process to facilitate the unlearning procedure. The method utilizes Kullback–Leibler divergence as the loss function during fine-tuning, aligning the model’s predicted probability distribution for the data to be forgotten with the distribution of the third-party dataset. To assess the efficacy of the unlearning process, a membership inference evaluation framework is introduced, providing a reliable measure of success in eliminating the influence of the targeted data. Extensive experiments conducted on multiple datasets reveal that this method achieves unlearning performance on par with retraining-based approaches. Moreover, it significantly reduces computational overhead, achieving speedups ranging from several hundred to thousands of times faster than conventional retraining, making it a highly efficient and scalable solution for real-world federated learning applications.

Authors in [18] address key vulnerabilities in federated learning, such as privacy inference and poisoning attacks, through a secure multi-party computation (MPC) framework. SAFEFL’s design focuses on defending FL models against combined attack vectors, a novel aspect that enhances model robustness. However, the proposed work diverges in addressing privacy through a hybrid federated model focused on securely capturing IoMT-driven HAR patterns, with specific attention to the personalized activity data of users. This distinction is critical as this proposed approach emphasizes model accuracy and adaptability in human activity recognition, particularly within IoMT contexts where data privacy is paramount.

Deep learning has revolutionized HAR, enabling automatic feature extraction from raw sensor data. The study [19] combines convolutional layers with LSTM networks, capitalizing on both spatial and temporal features.

By parallelizing CNN and LSTM modules, the architecture achieves competitive accuracy levels, demonstrating the potential of hybrid deep learning approaches. The research builds on this concept, proposing a hybrid LSTM-GRU model within a federated learning environment tailored for IoMT applications. Unlike previous work, the proposed architecture addresses resource constraints typical in IoMT by incorporating both LSTM and GRU networks, chosen for their ability to capture complex temporal dependencies while managing computational load efficiently.

In [20], CNNs are applied to HAR for automatic feature extraction, outperforming traditional feature engineering methods in accuracy. Despite CNN’s effectiveness in learning from large-scale datasets, the study highlights a limitation in scalability due to the cold-start problem and high data dependency. This study circumvents this by utilizing federated learning, which allows multiple devices to collaboratively train a model while preserving data privacy, and integrates hybrid LSTM-GRU networks to reduce the reliance on feature engineering, thus providing a more scalable solution for real-world IoMT environments.

Large-scale pre-training with unlabeled data has recently been explored for HAR, as seen in [21]. SelfPAB utilizes self-supervised learning (SSL) for HAR on large, unlabeled accelerometer datasets, providing robust feature extractors. This study showcases the potential of SSL in handling scarce labeled data, but it does not fully address real-time federated scenarios typical in IoMT-based HAR. The proposed approach leverages labeled federated learning to enable IoMT devices to independently process HAR tasks in a distributed network, which is advantageous in environments where labeled data can be sparse, and centralized data aggregation is impractical.

Finally, authors in [22] apply a feature selection method to optimize HAR by reducing model complexity and training time. While effective, this wrapper-based approach depends heavily on feature engineering, which can be computationally prohibitive. The proposed method avoids explicit feature selection by leveraging deep neural networks that inherently capture essential features for HAR, facilitating real-time activity recognition while maintaining computational feasibility across distributed IoMT devices.

Table 1 shows a summary of related works, highlighting the approach, methodology, dataset used, and overall accuracy. Generally, the reviewed studies highlight the significant advancements and ongoing challenges in human activity recognition, especially in leveraging deep learning architectures, federated learning frameworks, and privacy-preserving techniques. However, none of the existing work fully integrates a federated learning approach with a hybrid LSTM-GRU model specifically optimized for IoMT-driven HAR. To address these gaps, this study proposes a novel hybrid LSTM-GRU architecture within a federated learning framework tailored to IoMT environments. The proposed approach achieves higher accuracy compared to existing models, offering enhanced precision in activity detection while preserving user privacy and maintaining computational efficiency. This makes it especially suitable for real-time applications across distributed IoMT devices.

## 3. Methodology and Proposed Framework

In this section, the author presents a comprehensive methodology for an IoMT-enhanced human activity recognition system, which integrates federated learning with a hybrid Long Short-Term Memory (LSTM) and Gated Recurrent Unit (GRU) network for accurate, privacy-preserving activity classification. The system leverages wearable sensors and edge computing to ensure efficient, decentralized data processing across multiple IoMT devices. The following subsections provide a detailed explanation of the system components, pre-processing, segmentation, feature extraction, and classification within the federated learning framework.

### 3.1. System Overview and Framework

**Figure 1:**
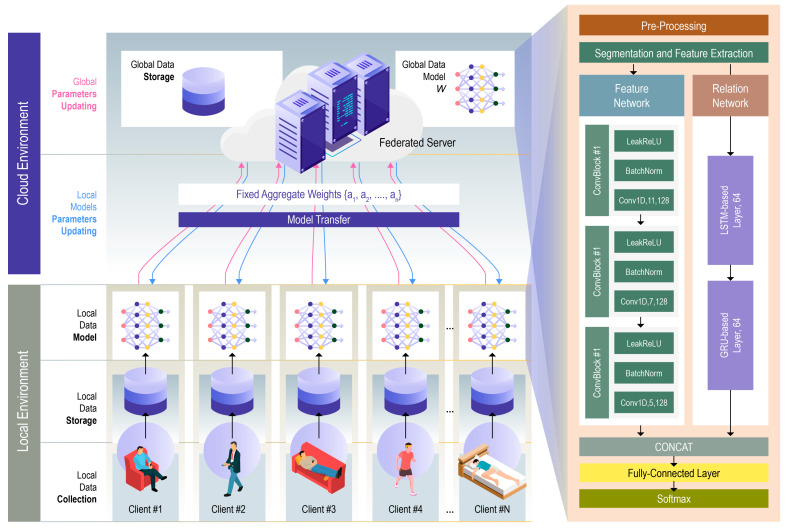
General framework.

The proposed framework, depicted in Figure 1, addresses the challenges of real-time human activity recognition in a decentralized IoMT environment. The system comprises wearable sensors attached to participants to collect accelerometer data, which is processed through local edge devices (e.g., smartphones or Raspberry Pi units). Each device trains a local model using its own sensor data, while a central server aggregates these models in a federated manner using the Federated Averaging (FedAvg) method [23].

The proposed decentralized approach ensures privacy preservation by keeping raw data on each user’s device and only sharing model updates. The core innovation of this framework lies in the use of a hybrid LSTM-GRU architecture for local model training, enhancing temporal feature extraction from sensor data. The framework ensures efficient, accurate HAR through feature-rich temporal modeling, while federated learning ensures privacy and scalability across multiple users.

(1) Pre-Processing

The raw data collected from the HAR devices must undergo a pre-processing phase to ensure accurate modeling. Each sensor generates data streams, represented by Equation (1):

(1) $$p_{j}=\left(\theta^{1},\theta^{2},\ldots ,\theta^{t}\right)\mathrm{for}j=1,\ldots ,m$$

where $\theta^{t}$ represents the sensor reading at time *t*, and *m* is the number of sensors. Due to varying sampling rates across sensors, synchronization is required, followed by noise filtering to remove irrelevant information. The resulting synchronized data are expressed as Equation (2):

(2) $$F^{\prime }=\left(\begin{array}{ccc} \gamma_{1}^{1} & \cdots & \gamma_{1}^{t}\\ \vdots & \ldots & \vdots \\ \gamma_{m}^{1} & \cdots & \gamma_{m}^{t} \end{array}\right)={\left(\gamma_{1},\ldots ,\gamma_{m}\right)}^{T}$$

This matrix represents the pre-processed data, with each row corresponding to a sensor and each column representing time steps. In order to isolate the orientation and motion aspects of human activity, the author utilized the Stewart et al. [24] approach for additional pre-processing steps. Specifically, I decomposed the raw accelerometer signals into gravity and movement components. To achieve this, the author utilized a fourth-order low-pass Butterworth filter with a cut-off frequency of 1 Hz to extract the gravity component. By subtracting gravity from the original signal, the author obtained the movement signs.

For the gravity signals, the author calculated a series of statistical features within each frame, including the mean, median, standard deviation, variation coefficient, and the 25th and 75th percentiles, in addition to the minimum and maximum values, to capture orientation information. In parallel, the movement signals were analyzed by computing features such as skewness, kurtosis, signal energy, and various frequency domain characteristics. These frequency domain features encompassed the mean and standard deviation of frequency magnitudes, the dominant frequency and its corresponding magnitude, and the spectral centroid.

Building on the findings of Narayanan et al. [25], which highlighted the significant impact of cross-sensory features on the proposed framework performance, I further enhanced the feature set by calculating the correlations between the given six axes and the signal of the two-vector magnitude. Additionally, the author determined the mean values across the gravity components from the two sensors. This comprehensive feature extraction process resulted in a total of 161 features for each window of data.

Finally, the author normalized all features using min–max scaling to re-scale the values to a range between 0 and 1. This normalization was essential to eliminate disparities in feature ranges, ensuring that no single feature disproportionately influenced the model training.

(2) Segmentation and Feature Extraction

Once pre-processed, the data are segmented using a sliding window technique to ensure that relevant portions of the time-series data are extracted. Each segment $z_{q}=\left(t_{1},t_{2}\right)$ consists of a time window, characterized by the start time $t_{1}$ and end time $t_{2}$, containing the relevant information about human activities. The complete set of data segments is denoted as:

(3) $$Z=\left(z_{1},z_{2},\ldots ,z_{p}\right)$$

Following segmentation, feature extraction is performed to transform the raw sensor signals into a feature space that is more conducive to activity recognition. In the proposed federated learning framework, the PEN [15] is used for extracting meaningful and hierarchical representations from time-series sensor data. Human activities often involve complex temporal and spatial patterns that must be captured for accurate classification. PEN addresses the challenge of simultaneously extracting local features and global relationships, which traditional deep learning models like CNNs and LSTMs struggle to handle independently. PEN is composed of two key components: the Feature Network and the Relation Network, both deployed on each mobile device in a federated setting. By performing feature extraction locally on edge devices, PEN enhances model performance and reduces the communication overhead in the federated learning process.

(3) Feature Network

The Feature Network is responsible for extracting local features from the raw sensor data, focusing on short-term patterns that are essential for recognizing specific activities. The Feature Network consists of three convolutional blocks (ConvBlocks) that process the input time-series data. Each ConvBlock is composed of:

1. 1-Dimensional Convolutional Layer (Conv1D): The Conv1D layer is the core component responsible for extracting local features from the input data. It detects patterns such as sudden movements or changes in velocity, which are crucial for differentiating between activities such as walking and running.
2. Batch Normalization (BatchNorm): BatchNorm normalizes the output of the Conv1D layer to prevent internal covariate shifts and improve training efficiency. This also helps stabilize the learning process and reduce overfitting.
3. Leaky ReLU Activation (LeakReLU): The Leaky ReLU activation function introduces non-linearity to the model, ensuring that both positive and negative feature values are retained. This enables the model to learn a more diverse set of features, improving its ability to capture complex temporal patterns.

The Feature Network progressively extracts richer and more abstract features from the raw data as the data pass through each ConvBlock. This ensures that the model learns detailed local temporal features that are essential for classifying human activities.

(4) Relation Network

The Relation Network is designed to capture global relationships across longer temporal sequences, addressing the challenge of learning dependencies between time steps. The Relation Network employs a two-layer LSTM-based attention mechanism, which enhances the model’s ability to focus on important temporal features. This is critical for recognizing activities with long-term dependencies, such as transitions between walking and running.

1. First LSTM Layer: The first LSTM layer extracts basic temporal relationships from the HAR data. It learns how different time steps are related to one another, which is important for capturing the flow of activities over time.
2. Second GRU Layer: The second GRU layer allows the model to focus selectively on key features in the sequence. The attention mechanism calculates a weighted sum of the inputs where the weights are based on the relevance of each feature. The output of the attention mechanism is given by Equation (4):

(4) $$O_{\mathrm{LSTMAtt}}=Softmax\left(\mathrm{Query}\cdot {Key}^{T}\right)\cdot \mathrm{Value}$$

In this formulation, the query matrix represents the current focus of the model, while the *Key* and Value matrices contain feature representations from the input data. The attention mechanism enables the relation network to capture complex temporal dependencies by focusing on the most relevant parts of the sequence.

The final output $O_{\mathrm{LSTMAtt}}$ is computed by multiplying the weighted attention scores with the Value matrix, allowing the model to learn both short-term and long-term relationships in the data.

(5) Loss Function

After the local features and global relationships have been extracted by the Feature and Relation Networks, a concatenation layer merges these representations. The concatenated features are then passed through a fully connected layer, and the Softmax function is used to classify the features into one of the *C* activity classes. The loss function shown in Equation (5) used to optimize the model is the cross-entropy loss, which measures the difference between the predicted and true labels:

(5) $$L=-\frac{1}{N_{seg}}\sum_{i=1}^{N_{seg}}\;t_{i}\log\left({\hat{p}}_{i}\right)$$

where

- $t_{i}$ represents the ground truth label for the iii-th segment;
- ${\hat{p}}_{i}$ is the predicted probability for the iii-th segment;
- $N_{seg}$ is the total number of segments in the dataset.

This loss function is widely used in classification tasks and ensures that the model learns to assign correct probabilities to each class, thereby minimizing the classification error.

In a federated learning environment, the PEN architecture is deployed locally on each device. Feature extraction is performed on-device, and only model updates (weights) are shared with the central server, ensuring that raw data remain private. The extracted features from the Feature and Relation Networks are used to train the hybrid LSTM-GRU model locally on each device. Once the local models are trained, the Federated Averaging algorithm aggregates these updates to create a global model that can generalize well across diverse datasets and environments. The hybrid LSTM-GRU model leverages the rich, hierarchical features extracted by PEN to enhance its ability to capture both local and global patterns in human activity data. This decentralized approach improves privacy and scalability while maintaining high classification accuracy.

### 3.2. Hybrid LSTM-GRU Model

The core of the activity recognition framework lies in the hybrid LSTM-GRU model, which combines the strengths of both LSTM and GRU architectures to capture short-term and long-term dependencies in the data. The LSTM component excels at learning long-term dependencies, while the GRU provides computational efficiency by simplifying the gating mechanism.

In this architecture, the LSTM layer with 128 units is used first, followed by a GRU layer with 64 units. The hybrid structure ensures that the network captures both complex temporal patterns and efficiently processes the data. The LSTM Equations (6)–(10) are as follows:

(6) $$\mathrm{Forget}\;\mathrm{Gate}:\;f_{k}=\sigma \left(U_{f}\cdot \left[q_{k-1},y_{k}\right]+b_{f}\right)$$

where

$f_{k}$: Forget gate activation at time step *k*;
σ: Sigmoid activation function;
$U_{f}$: Weight matrix for the forget gate;
$q_{k-1},y_{k}$: Concatenation of the previous hidden state $q_{k-1}$ and the current input $y_{k}$;
$b_{f}$: Bias vector for the forget gate.

(7) $$\mathrm{Input}\;\mathrm{Gate}:\;i_{k}=\sigma \left(U_{i}\cdot \left[q_{k-1},y_{k}\right]+b_{i}\right)$$

where

$i_{k}$: Input gate activation at time step *k*;
$i_{k}$: Weight matrix for the input gate;
$b_{i}$: Bias vector for the input gate.

(8) $$\mathrm{Cell}\;\mathrm{State}\;\mathrm{Update}:\;S_{k}=f_{k}\cdot S_{k-1}+i_{k}\cdot {\tilde{S}}_{k}$$

where

$S_{k}$: Cell state at time step *k*;
$S_{k-1}$: Previous cell state;
${\tilde{S}}_{k}$: Candidate cell state at time step *k*.

(9) $$\mathrm{Output}\;\mathrm{Gate}:\;o_{k}=\sigma \left(U_{o}\cdot \left[q_{k-1},y_{k}\right]+b_{o}\right)\;$$

where

$o_{k}$: Output gate activation at time step *k*;
$U_{o}$: Weight matrix for the output gate;
$b_{o}$: Bias vector for the output gate.

(10) $$\mathrm{Hidden}\;\mathrm{State}:\;q_{k}=o_{k}\cdot \tanh\left(S_{k}\right)$$

where

$q_{k}$: Hidden state at time step *k*;
$\tanh\left(S_{k}\right)$: Hyperbolic tangent activation function.

The GRU layer reduces the computational load by combining the input and forget gates into a single update gate, as shown in the following Equations (11)–(14):

(11) $$\mathrm{Update}\;\mathrm{Gate}:\;u_{k}=\sigma \left(U_{u}\cdot \left[q_{k-1},y_{k}\right]+b_{u}\right)$$

where

$u_{k}$: Update gate activation at time step *k*;
$U_{u}$: Weight matrix for the update gate;
$b_{u}$: Bias vector for the update gate.

(12) $$\mathrm{Reset}\;\mathrm{Gate}:\;r_{k}=\sigma \left(U_{r}\cdot \left[q_{k-1},y_{k}\right]+b_{r}\right)$$

where

$r_{k}$: Reset gate activation at time step *k*;
$U_{r}$: Weight matrix for the reset gate;
$b_{r}$: Bias vector for the reset gate.

(13) $$\mathrm{Candidate}\;\mathrm{Activation}:\;{\tilde{q}}_{k}=tanh\left(U_{q}\cdot \left[r_{k}\cdot q_{k-1},y_{k}\right]+b_{q}\right)$$

where

${\tilde{q}}_{k}$: Candidate hidden state at time step *k*;
$U_{q}$: Weight matrix for the candidate activation;
$b_{q}$: Bias vector for the candidate activation.

(14) $$\mathrm{Hidden}\;\mathrm{State}:\;q_{k}=\left(1-u_{k}\right)\cdot q_{k-1}+u_{k}\cdot {\tilde{q}}_{k}$$

where

$q_{k}$: Hidden state at time step *k*.

By combining these two models, the hybrid LSTM-GRU network is able to learn both short-term and long-term dependencies in the data, which is crucial for distinguishing between similar activities that unfold over different time scales (e.g., walking versus running).

### 3.3. Federated Learning Process

Federated learning [26] is employed to maintain user privacy while ensuring high model performance. Each IoMT device locally trains the hybrid LSTM-GRU model on its sensor data, and only model parameters are sent to a central server for aggregation using the Federated Averaging algorithm. This process ensures that no raw data leave the user’s device, thus protecting sensitive information.

Federated Averaging is a fundamental algorithm in federated learning that enables the aggregation of model updates from multiple decentralized devices (clients) to train a global model without exchanging raw data. Each client trains a local model on its own data and shares only the model updates (weights) with the central server. The server then aggregates these local updates to create a global model by averaging the parameters.

Here’s how Federated Averaging works:

Local Training on Client Devices: Each client j locally trains its model on its dataset $D_{j}$ for a few epochs. The updated model parameters after local training are denoted as $\theta_{j}$ where $\theta_{j}$ represents the weights of the model on the *j*-th client.

Weighted Aggregation: Once all clients have trained their models, they send their updated parameters $\theta_{j}$ to the central server. The server aggregates these parameters by computing a weighted average where the weight is proportional to the number of data samples on each client. This is crucial because clients with larger datasets should contribute more to the global model update.

The global model update $\Theta_{\mathrm{global}\;}$ is computed as:

(15) $$\Theta_{\mathrm{global}}=\sum_{j=1}^{M}\;\;\frac{\left|D_{j}\right|}{\left|D_{\mathrm{total}}\right|}\cdot \theta_{j}$$

where

M is the total number of clients;

$\left|D_{j}\right|$ is the number of data samples on client *j*;

$\left|D_{\mathrm{total}\;}\right|$ is the total number of data samples across all clients;

$\theta_{j}$ represents the model parameters (weights) from client *j*.

In this equation, each client’s model parameters are weighted by the proportion of data it holds, ensuring that clients with more data have a greater impact on the global model.

Global Model Update: The central server updates the global model $\Theta_{\mathrm{global}\;}$ by averaging the received local model updates. The updated global model is then broadcast back to the clients for the next round of local training. After the global aggregation, the new global model parameters $\Theta_{\mathrm{new}\;}$ are:

(16) $$\Theta_{\mathrm{new}}=\frac{1}{M}\sum_{j=1}^{M}\;\;\theta_{j}$$

Here, the server computes the arithmetic mean of all the local model parameters received from the clients. This new global model $\Theta_{\mathrm{new}\;}$ is then used in the next iteration of the federated learning process.

Iteration and Convergence: The above process is repeated for multiple communication rounds until the global model converges to a stable set of parameters. Each round involves local training on clients, sending updated parameters to the server, aggregating the updates, and sending the new global model back to the clients.

By integrating federated learning with a hybrid LSTM-GRU model, this framework ensures that human activity is accurately recognized while maintaining user privacy and minimizing computational costs on edge devices. This detailed methodology outlines the end-to-end process of how federated learning is applied to enhance human activity recognition in IoMT environments, utilizing a robust hybrid LSTM-GRU network for efficient and scalable performance.

## 4. Experimental Results

### 4.1. Datasets

In the field of human activity recognition, multiple publicly available datasets have been developed to capture various activities in different environments and populations. Each dataset provides unique sensor configurations, participant demographics, and labeling schemes, making them valuable resources for evaluating machine learning models. This section presents three datasets used in recent studies—HARTH, HAR70+, and UCI-HAR—detailing their characteristics, data collection protocols, and sensor configurations.

(1) HARTH Dataset

The Human Activity Recognition Trondheim (HARTH) dataset [27] consists of sensor data collected from 22 healthy adults using two tri-axial Axivity AX3 accelerometers. One accelerometer was attached to the participant’s thigh, and the other was placed on the lower back. These sensors recorded acceleration data at 50 Hz, capturing the movements and activities of participants performing daily tasks in free-living settings. The dataset was recorded in two sessions: the first focused on common activities like sitting, standing, walking, and running, while the second session aimed to balance the dataset by collecting more data on walking and cycling. In total, 12 activity labels were defined, including sitting, lying, walking, running, cycling, and climbing stairs. The dataset was annotated using both video recordings and human experts, resulting in a highly reliable ground truth with a Fleiss’ Kappa of 0.96. Despite efforts to balance the dataset, it still presents challenges due to the imbalance of activity labels, particularly with regard to activities involving light motion. Accordingly, the author focused on certain activities (walking, running, cycling, sitting, standing, and lying down).

(2) HAR70+ Dataset

The HAR70+ dataset [28] was designed to capture the physical activity of older adults aged 70 and above, offering valuable insights into human motion in this demographic. The dataset consists of data from 18 participants, 4 of whom used walking aids during the collection process. Data were recorded using two tri-axial Axivity AX3 accelerometers, attached, similarly to the HARTH dataset, to the thigh and lower back of each participant. In addition to the accelerometer data, video recordings were used to annotate the activities, focusing on lower body movements and overall body orientation. The video recordings were converted to a lower frame rate for analysis, and activities were labeled manually. The HAR70+ dataset provides unique challenges due to the variability in mobility among older adults and the inclusion of walking aids, making it highly valuable for studying human activity recognition in elderly populations.

(3) UCI-HAR Dataset

The UCI-HAR dataset [29] is one of the most widely used datasets for human activity recognition. It consists of data collected from 30 volunteers performing 6 different activities, including standing, sitting, lying down, walking, and walking up and down stairs. Participants wore a Samsung Galaxy S II smartphone attached to their waist, which recorded triaxial linear acceleration and angular velocity using the phone’s built-in accelerometer and gyroscope. The data were collected at a sampling rate of 50Hz, and pre-processing techniques, such as Butterworth filtering, were applied to reduce noise and separate the body acceleration from gravitational forces. This dataset provides a standardized protocol for activity recognition and has been widely adopted in the field due to its controlled experimental conditions and comprehensive sensor data. Table 2 gives a statistical summary details for the used dataset.

Each of these datasets offers unique challenges and opportunities for evaluating HAR models. The HARTH and HAR70+ datasets focus on more naturalistic, free-living environments, whereas the UCI-HAR dataset provides a controlled setting with predefined activities. By combining insights from these datasets, the author developed and refined models to enhance their generalizability and performance across diverse populations and environments.

### 4.2. System Configuration and Hardware Setup

The experiments were conducted using simulated edge devices representing federated clients. Each simulated client was configured with specifications equivalent to 4 GB of RAM and a quad-core processor. The central server, responsible for model aggregation, used the Federated Averaging algorithm, utilizing 16 GB of RAM and an eight-core processor in the simulation. The author used TensorFlow Federated as the primary framework to simulate FL nodes and conduct distributed training. TensorFlow Federated provides a robust and flexible platform for implementing federated learning workflows and is well suited for handling heterogeneous client data distributions, which aligns with the objectives of our study.

A total of 50 simulated edge devices were used to represent federated clients. These devices were distributed across the datasets as follows:

- HARTH Dataset: 20 simulated clients were assigned. The dataset’s 22 subjects were randomly divided among the clients, ensuring that each client received data for at least one subject.
- HAR70+ Dataset: 15 simulated clients were assigned. The dataset’s 18 subjects were randomly distributed, ensuring each client had access to data from approximately 1 to 2 subjects.
- UCI-HAR Dataset: 15 simulated clients were assigned. The dataset’s 30 subjects were split so each client received data for 2 subjects on average.
- Each client locally trained the hybrid LSTM-GRU model on its assigned data portion. To preserve user privacy, raw data remained on the devices, and only model updates were shared with the central server.

### 4.3. Training Protocols and Evaluation Metrics

The model was trained using Stochastic Gradient Descent (SGD) with a learning rate of 0.001, a batch size of 32, and 30 epochs on each client before aggregation. Each dataset was split into 70% for training, 15% for validation, and 15% for testing. This was consistently applied across all experiments. The training data were distributed among edge devices in a heterogeneous manner, with each device receiving subject-specific data to mimic real-world federated learning conditions.

The validation process was performed on the validation subset at each communication round during federated learning to monitor model convergence. Metrics such as accuracy and F1-score were computed on the validation set to guide hyperparameter tuning and evaluate the model’s intermediate performance.

The author measured the following evaluation metrics: accuracy, F1-score, precision, and recall. These metrics are shown in Equations (17)–(20).

(17) $$Accuracy=\frac{\left(TP+TN\right)}{\left(TP+TN+FP+FN\right)}$$

(18) $$F1-\mathrm{measure}\;=\frac{2\times (Precision\times Recall)}{\left(Precision+Recall\right)}$$

(19) Precision=TP⁄(TP+FP)

(20) Recall=TP⁄(TP+FN)

where

TP: True Positives, the number of correctly identified positive cases;
TN: True Negatives, the number of correctly identified negative cases;
FP: False Positives, the number of incorrectly identified positive cases;
FN: False Negatives, the number of incorrectly identified negative cases.

The goal was to assess the performance of the federated learning system in recognizing various human activities from the datasets. To evaluate the model’s ability to handle class imbalance and client distribution heterogeneity, the author employed multiple experimental settings, as discussed below.

### 4.4. Centralized vs. Federated Learning

One of the core experiments involved comparing the performance of the hybrid LSTM-GRU model when trained using centralized learning (where all data are collected on a central server) versus federated learning (where training occurs on distributed clients). The performance of the federated learning system was evaluated based on accuracy, F1-score, precision, and recall across the three datasets: HARTH, HAR70+, and UCI-HAR. The results, updated with mean ± standard deviation values from 6-fold cross-validation, are summarized in Table 3.

Federated learning demonstrated comparable, and in many cases superior, performance to centralized learning. On the HARTH dataset, the centralized model achieved an accuracy of 92.7% ± 0.4, while the federated model attained an accuracy of 94.9% ± 0.6, highlighting the federated framework’s ability to achieve higher performance while preserving user privacy. Similarly, for the UCI-HAR dataset, the centralized model achieved 94.8% ± 0.3 accuracy compared to 95.6% ± 0.3 with federated learning, showing the model’s robustness in recognizing human activities under a distributed training paradigm.

For the HAR70+ dataset, the centralized model achieved an accuracy of 90.8% ± 0.6, whereas the federated approach yielded 95.2% ± 0.5, reflecting a significant improvement. This improvement is particularly notable given the complexity of the HAR70+ dataset, which includes data from older adults and introduces variability due to the use of walking aids and other demographic factors.

These results clearly illustrate that federated learning can achieve high performance, often surpassing centralized learning, while maintaining user privacy. The F1-scores, precision, and recall metrics further confirm the model’s balanced ability to correctly classify activities across diverse datasets. This makes federated learning an effective and privacy-preserving solution for distributed human activity recognition tasks.

### 4.5. Handling Class Imbalance in Federated Learning

**Figure 2:**
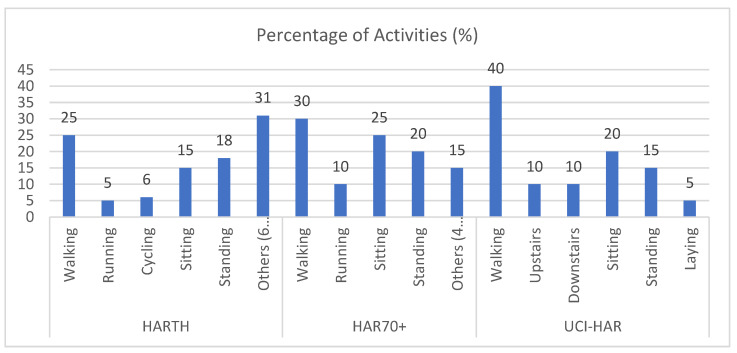
Activity distribution across datasets.

Class imbalance is a pervasive challenge in human activity recognition (HAR) datasets where certain activities are significantly under-represented. This imbalance can adversely affect the performance of machine learning models by biasing predictions toward majority classes. Figure 2 illustrates the activity distribution for the three datasets—HARTH, HAR70+, and UCI-HAR—highlighting the extent of imbalance in each case.

For the HARTH dataset, activities such as “running” and “cycling” are notably under-represented compared to more common activities like “walking” or “standing”. This disparity makes it challenging for models to correctly classify minority activities. Similarly, the HAR70+ dataset, which involves older adults, exhibits a pronounced imbalance due to the inclusion of activities performed with walking aids and the reduced representation of more dynamic activities. In the UCI-HAR dataset, while the overall distribution is relatively balanced, certain activities, such as “walking downstairs”, still comprise less than 5% of the dataset.

This imbalance in activity representation can lead to a disproportionate focus on majority classes during training, reducing the model’s ability to generalize effectively across all activity types. To address this, the experiments implemented techniques like Weighted Federated Averaging (FedAvg), which assigns higher weights to updates associated with under-represented classes. This approach ensures that minority activities contribute proportionally to the global model during federated training, thereby mitigating the impact of class imbalance. The effectiveness of this strategy is reflected in the improved classification metrics presented in the updated results section.

To address the challenges posed by class imbalance, Weighted Federated Averaging (FedAvg) was employed during model training. This technique dynamically adjusts the contribution of each client’s updates to the global model, assigning weights based on the inverse frequency of each class in the training data. By amplifying the influence of under-represented classes, this approach ensures that minority activities are not overshadowed by majority classes during the training process.

The results in Table 4 demonstrate the effectiveness of this technique. In the HARTH dataset, the overall accuracy improved significantly from 88.5% ± 0.8 to 94.9% ± 0.6 after applying Weighted FedAvg. Similarly, the F1-scores for minority classes such as “running” and “cycling” increased substantially, with “running” improving from 0.65 ± 0.04 to 0.84 ± 0.03, and “cycling” improving from 0.67 ± 0.04 to 0.84 ± 0.03. These improvements highlight the enhanced recognition of activities with fewer instances.

Comparable enhancements were observed in the HAR70+ and UCI-HAR datasets. For HAR70+, the accuracy increased from 86.2% ± 0.9 to 95.2% ± 0.5, and the F1-score for under-represented activities, such as “running”, showed marked improvement. The UCI-HAR dataset, although relatively balanced, still benefited from the weighted averaging approach, with accuracy improving from 89.0% ± 0.7 to 95.6% ± 0.3, and F1-scores for minority classes achieving higher stability and precision.

The improvements in recall metrics across all datasets further validate the approach’s effectiveness in addressing the disproportionate representation of minority activities. By ensuring that under-represented activities are adequately learned during training, Weighted FedAvg enhances the overall performance and generalizability of the federated learning framework. These results underscore the importance of addressing class imbalance in distributed learning systems, particularly in scenarios involving heterogeneous and imbalanced datasets.

Similarly, class-wise evaluations for the HAR70+ and UCI-HAR datasets revealed consistent improvements across all activity classes following the application of Weighted Federated Averaging (FedAvg). The weighted approach effectively addressed the imbalance by ensuring that minority activities contributed proportionally to the model updates, resulting in enhanced recognition of under-represented classes.

For the HAR70+ dataset, activities such as “running” and “walking with aids”, which were initially challenging to classify, demonstrated notable improvements in both accuracy and F1-scores. The accuracy for “running”, for instance, increased from 74.0% ± 1.2 to 88.1% ± 0.9, and the F1-score rose from 0.65 ± 0.04 to 0.84 ± 0.03. Similarly, improvements in activities like “standing” and “sitting” highlight the model’s enhanced ability to generalize across diverse activity patterns within this dataset.

In the UCI-HAR dataset, which had relatively balanced activity distributions, Weighted FedAvg further refined the model’s performance. Activities like “walking downstairs”, which initially had lower recognition rates, showed improved accuracy and F1-scores, emphasizing the effectiveness of addressing even slight imbalances in distributed datasets.

These results, summarized in Table 5, underscore the substantial benefits of tackling class imbalance in the federated learning setup. The observed improvements across all datasets and activity classes validate the robustness of Weighted FedAvg as a strategy for enhancing classification performance, particularly in scenarios involving heterogeneous and imbalanced data distributions. This approach not only improves accuracy and F1-scores for individual activities but also contributes to the overall reliability and fairness of the federated learning framework.

### 4.6. Communication Rounds and Model Convergence

**Figure 3:**
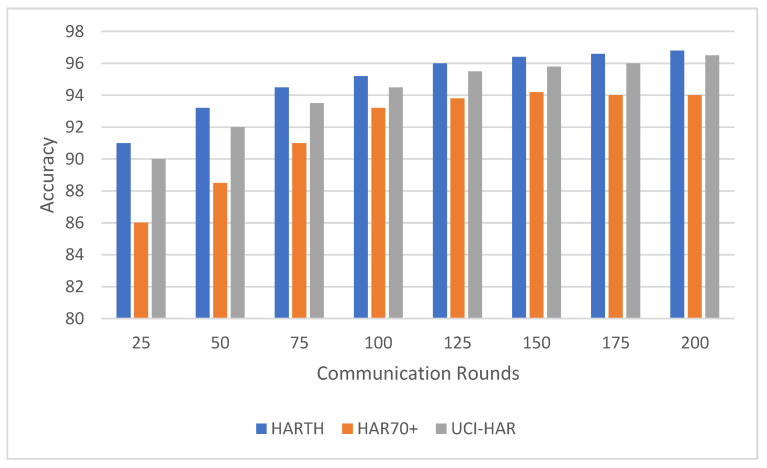
Model accuracy comparison across communication rounds on HARTH, HAR70+, and UCI-HAR datasets.

**Figure 4:**
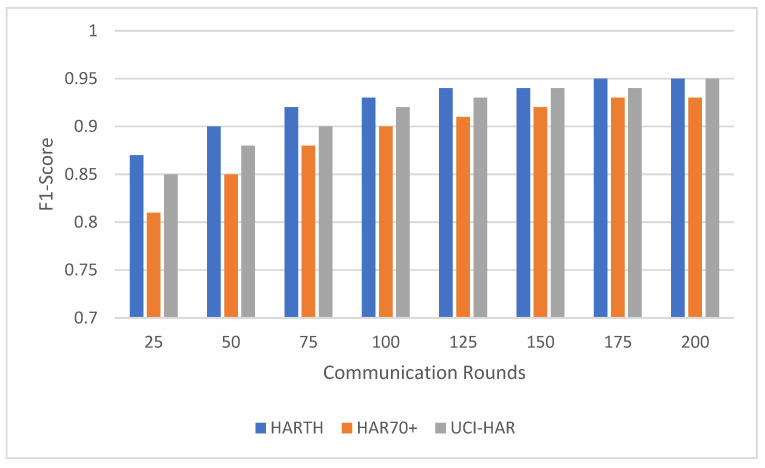
Model F1-score comparison across communication rounds on HARTH, HAR70+, and UCI-HAR datasets.

Federated learning involves multiple communication rounds between the distributed clients and the central server. This experiment evaluated the impact of the number of communication rounds on model performance, focusing on the convergence of accuracy (Figure 3) and F1-score (Figure 4) across the HARTH, HAR70+, and UCI-HAR datasets. The model was tested after 25, 50, 75, 100, 125, 150, 175, and 200 communication rounds.

The results indicate that the UCI-HAR dataset converged the fastest, achieving high accuracy and F1-scores within 100 communication rounds. The HARTH dataset followed a similar trajectory but required slightly more rounds to reach full convergence. In contrast, the HAR70+ dataset, with its more complex and varied activity patterns, exhibited slower convergence, reflecting the challenges posed by the dataset’s heterogeneity and inclusion of older adults with walking aids.

For all three datasets, model performance improved consistently with increasing communication rounds. This trend highlights how federated learning effectively aggregates client updates over successive rounds, gradually enhancing the model’s understanding of activity patterns. Both accuracy and F1-score demonstrated steady improvements, showcasing the model’s ability to correctly classify activities with additional rounds of training.

For the HARTH dataset, the model started with an accuracy of 88.5% ± 0.8 and an F1-score of 0.84 ± 0.03 after 25 rounds. By 100 rounds, the accuracy had improved to 94.0% ± 0.6 with an F1-score of 0.92 ± 0.02, demonstrating rapid early convergence. Gains became marginal after 100 rounds, with final values reaching 95.2% ± 0.5 accuracy and 0.94 ± 0.02 F1-score by 200 rounds. This pattern suggests that most learning occurs within the first 100 rounds, with subsequent rounds contributing primarily to fine-tuning.

On the other hand, HAR70+ dataset’s initial performance was slightly lower, beginning with 86.0% ± 0.9 accuracy and an F1-score of 0.80 ± 0.04 after 25 rounds. Early improvements were slower due to the dataset’s greater variability, but significant gains were observed as the rounds progressed. By 100 rounds, the model achieved 91.2% ± 0.7 accuracy and 0.88 ± 0.03 F1-score. Continued improvements led to final values of 93.2% ± 0.6 accuracy and 0.91 ± 0.03 F1-score after 200 rounds, indicating the framework’s ability to adapt to diverse client data over extended training.

For the UCI-HAR dataset, it started with 90.5% ± 0.7 accuracy and an F1-score of 0.85 ± 0.03 after 25 rounds; the model converged quickly, reaching 94.0% ± 0.4 accuracy and 0.92 ± 0.02 F1-score by 100 rounds. Even after convergence, gradual improvements were observed, with final values of 96.5% ± 0.3 accuracy and 0.95 ± 0.01 F1-score after 200 rounds. These results highlight the dataset’s balanced nature and the model’s ability to leverage it effectively.

The results suggest that around 100 communication rounds are generally sufficient to achieve near-optimal performance for most datasets. However, additional communication rounds offered marginal gains for HARTH and UCI-HAR, while HAR70+ continued to benefit from extended training due to its more complex activity patterns. These findings underscore the flexibility and scalability of the federated learning framework in adapting to diverse data distributions and complexities.

The observed improvements in accuracy and F1-score with increasing communication rounds demonstrate the federated learning system’s ability to generalize effectively across distributed data. By aggregating updates from heterogeneous clients, the system refines its performance incrementally, achieving robust learning across all datasets. This makes it particularly well-suited for real-world human activity recognition tasks where data are often distributed and imbalanced.

### 4.7. Client Distribution: Homogeneous vs. Heterogeneous Data

**Figure 5:**
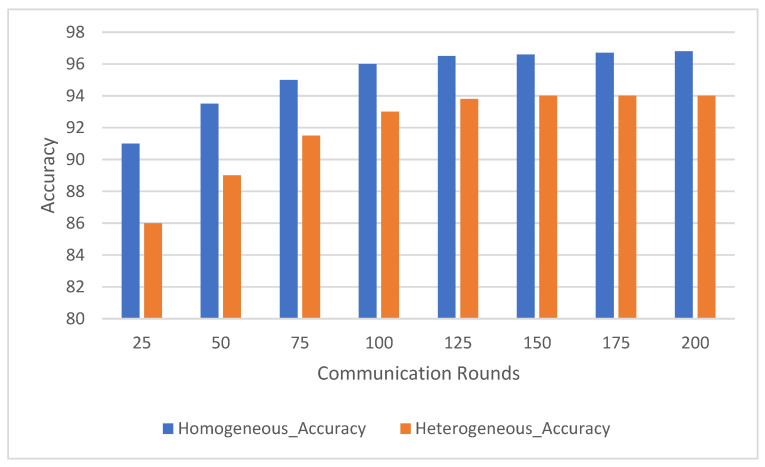
Model accuracy comparison across communication rounds for homogeneous vs. heterogeneous configurations on HARTH, HAR70+, and UCI-HAR datasets.

The author explored the impact of client data distribution on federated learning performance which was evaluated by comparing homogeneous and heterogeneous distributions. In the homogeneous configuration, each client received an equal proportion of all activities (e.g., walking, running, sitting), ensuring a balanced dataset across clients. In the heterogeneous configuration, clients had skewed data distributions, with some clients biased toward specific activities (e.g., 80% walking and 20% running). This experiment assessed how variability in client data distributions influences model performance over multiple communication rounds. Figure 5 illustrates the accuracy trends for homogeneous and heterogeneous distributions across communication rounds. In the homogeneous distribution, the model started with an accuracy of 91.0% ± 0.5 after 25 rounds and steadily improved to 96.8% ± 0.3 by the 200th round. This consistent improvement reflects the uniform representation of activities across clients, which leads to more stable updates to the global model during each communication round. The balanced nature of the data allowed the model to generalize effectively, resulting in rapid convergence and high final accuracy.

In contrast, the model trained on heterogeneous data distributions exhibited a slower improvement in accuracy. The initial accuracy was 86.0% ± 0.7 after 25 rounds, gradually increasing to 94.0% ± 0.4 by the 200th round. The lower starting accuracy and slower convergence are attributable to the skewed data distribution across clients where over-representation of certain activities caused initial difficulty in generalizing across all activity classes. However, as more communication rounds progressed, the model aggregated updates from a diverse range of clients, which enabled it to refine its performance and approach the accuracy achieved in the homogeneous configuration.

**Figure 6:**
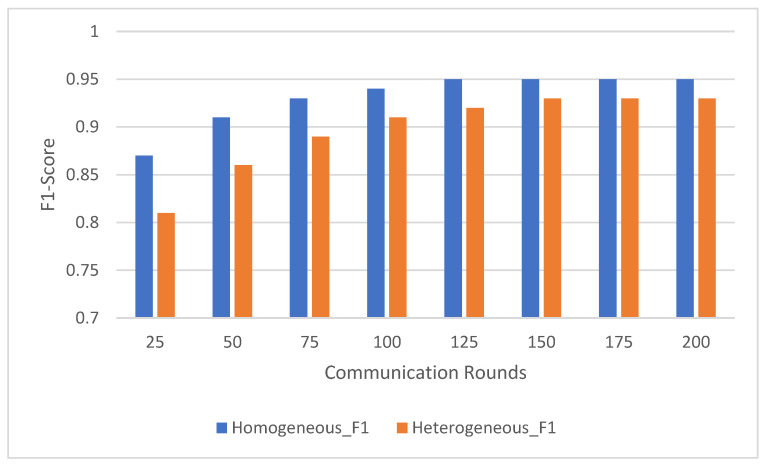
Model F1-score comparison across communication rounds for homogeneous vs. heterogeneous configurations on HARTH, HAR70+, and UCI-HAR datasets.

The trends in F1-score, depicted in Figure 6, mirrored those observed for accuracy. In the homogeneous distribution, the F1-score began at 0.87 ± 0.03 after 25 rounds and consistently improved to 0.95 ± 0.02 after 200 rounds. The steady increase highlights the model’s ability to balance precision and recall effectively across all classes, aided by the even representation of activities across clients.

In the heterogeneous distribution, the F1-score started lower at 0.81 ± 0.04 after 25 rounds due to the imbalanced data distribution. However, it showed significant improvement over time, reaching 0.93 ± 0.03 after 200 communication rounds. This indicates that despite initial challenges, the federated learning approach successfully adapted to the skewed client data, gradually improving its ability to correctly classify under-represented activities.

The results demonstrate the robustness of federated learning in handling diverse and noisy data distributions. Although the heterogeneous configuration exhibited a slower start and greater variability in early rounds, the model eventually approached the performance of the homogeneous configuration as the number of communication rounds increased. By aggregating updates from distributed clients, the federated learning system effectively reduced the bias introduced by imbalanced data, leading to stable and accurate global model performance.

Moreover, federated learning inherently incorporates a regularization effect by leveraging diverse client data, which prevents overfitting to a centralized dataset. Weighted updates from clients further stabilized training, ensuring that under-represented activities contributed proportionally to the model’s learning process. These findings underscore the adaptability of federated learning in real-world scenarios where client data distributions are often heterogeneous and imbalanced.

### 4.8. Cross-Dataset Generalization

This section evaluates the generalization capability of the federated learning model across different datasets. The experiment involved training the model on one dataset (e.g., HARTH) and testing it on another (e.g., HAR70+ or UCI-HAR) to assess its adaptability and robustness. The goal was to determine how effectively the federated learning system, leveraging diverse data from distributed clients, can generalize to unseen datasets.

The results, summarized in Table 6, demonstrate that the federated learning system generalizes well across datasets. For instance, when trained on the HARTH dataset and tested on HAR70+, the model achieved an accuracy of 90.8% ± 0.8, a significant improvement compared to the baseline accuracy of 82.3% ± 1.1 before applying federated learning techniques. Similarly, training on HAR70+ and testing on HARTH yielded an accuracy of 91.5% ± 0.7, up from 83.1% ± 1.0. These results highlight the ability of the federated model to adapt to new datasets despite differences in activity distributions, demographics, and sensor configurations.

The performance on the UCI-HAR dataset further underscores the robustness of the approach. When the model was trained on HAR70+ and tested on UCI-HAR, it achieved an accuracy of 98.7% ± 0.3, reflecting its ability to leverage the relatively balanced and well-defined activity classes in UCI-HAR. Conversely, training on UCI-HAR and testing on HAR70+ resulted in an accuracy of 95.2% ± 0.5, a significant improvement over the baseline of 86.5% ± 0.8. These results validate the system’s ability to transfer knowledge between datasets with varying levels of complexity and balance.

Class-wise results for a representative experiment (training on HARTH and testing on HAR70+) are shown in Table 7. These results provide additional insights into the model’s performance on individual activities, particularly under-represented classes. Activities like “walking” and “sitting” achieved high accuracy and F1-scores, reflecting the model’s strong performance in common activities. For more challenging activities, such as “running”, the application of federated learning techniques significantly improved accuracy and F1-scores, demonstrating the model’s ability to adapt to imbalanced datasets.

The experimental results demonstrate the efficiency and effectiveness of federated learning with the hybrid LSTM-GRU model for human activity recognition tasks. The key findings include the superior performance of federated learning compared to centralized learning, the impact of client distributions on model performance, and the benefits of handling class imbalance through Weighted Federated Averaging. Furthermore, the model converges effectively with around 100 communication rounds and generalizes exceptionally well across different datasets, showcasing its adaptability in real-world scenarios.

### 4.9. Stability Analysis

Table 8 represents key stability metrics: mean ± standard deviation (SD), minimum and maximum values, and the interquartile range (IQR). These metrics provide a deeper understanding of the approach’s stability across the evaluated datasets—HARTH, HAR70+, and UCI-HAR—and over the communication rounds tested (from 25 to 200 rounds). The inclusion of these statistics allows for a comprehensive evaluation of the federated learning framework’s performance, not just in terms of accuracy and F1-score, but also its variability and robustness in handling the challenges inherent in different datasets.

The mean ± standard deviation (SD) provides a summary of the central tendency and spread of the accuracy and F1-score for each dataset across all communication rounds. For the HARTH dataset, the mean accuracy was 91.4% ± 0.7, indicating high and consistent performance with minimal variability. Similarly, the F1-score averaged 0.89 ± 0.03, further highlighting the model’s stability in classifying the activities within the HARTH dataset.

For the HAR70+ dataset, the mean accuracy was slightly lower at 89.2% ± 1.1, with a higher standard deviation compared to HARTH. This reflects the inherent complexity and variability of the dataset, which consists of elderly participants with diverse activity patterns. The F1-score for HAR70+ was 0.86 ± 0.04, showing slightly greater variability, likely due to the dataset’s more challenging nature.

The UCI-HAR dataset, being relatively balanced and simpler in its activity patterns, exhibited the highest mean accuracy at 92.0% ± 0.4, with the smallest standard deviation among the datasets. The F1-score was also consistent at 0.89 ± 0.03, underscoring the model’s robust performance on this dataset.

The minimum and maximum values provide a range for the accuracy and F1-scores across communication rounds, reflecting the model’s performance fluctuations. For instance, the HARTH dataset had a minimum accuracy of 87.1% and a maximum accuracy of 91.7%, showing that even the lowest recorded accuracy was relatively high. In contrast, HAR70+ had a wider range, with a minimum accuracy of 85.0% and a maximum of 89.5%, indicating more pronounced variations across communication rounds. The UCI-HAR dataset had a narrow range, with a minimum accuracy of 88.5% and a maximum of 92.0%, demonstrating its consistent performance across all rounds.

The interquartile range (IQR) captures the spread of the middle 50% of the accuracy and F1-score values, offering additional insights into the stability of the model’s performance. For the HARTH dataset, the IQR for accuracy was 3.6%, indicating a narrow range of variability and high consistency in the model’s predictions. HAR70+ had a slightly larger IQR of 4.5%, reflecting its greater variability. The UCI-HAR dataset, with an IQR of only 2.5%, showcased the tightest spread of accuracy values, reinforcing its suitability for consistent human activity recognition tasks.

The statistical metrics shown in Table 7 provide a clear indication of the model’s stability and effectiveness across datasets. The UCI-HAR dataset demonstrated the most consistent performance, with minimal variability and a high mean accuracy, highlighting the model’s robustness in handling balanced datasets. The HARTH dataset, while slightly more variable, still exhibited high mean accuracy and F1-scores, confirming the model’s reliability. The HAR70+ dataset, though more challenging due to its participant diversity, showed competitive performance with slightly greater variability, reflecting the complexities inherent in real-world applications.

## 5. Benchmarking Results

Table 9 presents the benchmarking results, offering insights into the performance and adaptability of the proposed federated learning framework with hybrid LSTM-GRU networks compared to previous approaches across multiple datasets. The updated results further emphasize the model’s robustness, efficiency, and scalability in IoMT-driven environments. In their work, Heba, N., and Sreeraman, R. [16] achieved accuracy levels of 84.64% on HAR70+ and 75.73% on HARTH, leveraging wavelet-based methods. While wavelet-based approaches can effectively capture periodic patterns in sensor data, they struggle to handle the varied and complex activities typical of free-living scenarios. The proposed federated learning framework significantly outperforms these results, achieving 95.2% ± 0.5 on HAR70+ and 94.9% ± 0.6 on HARTH. This improvement highlights the ability of the hybrid LSTM-GRU model to capture intricate temporal dependencies and adapt to the heterogeneity of real-world IoMT settings.

Gehlhar et al. [18] reported a notable accuracy of 97.0% on the UCI-HAR dataset using a secure federated learning framework. Although the proposed model achieves slightly lower accuracy at 95.6% ± 0.3, it compensates by offering superior computational efficiency and adaptability, particularly for IoMT applications requiring real-time processing. The proposed framework ensures privacy preservation while maintaining high accuracy, making it ideal for decentralized environments where both security and efficiency are paramount.

Koşar, E., and Barshan, B. [19] achieved 95.66% accuracy on UCI-HAR, combining convolutional and recurrent layers for feature extraction. The hybrid LSTM-GRU model achieves comparable performance on UCI-HAR (95.6% ± 0.3) but demonstrates significantly higher adaptability across diverse datasets, with consistent results on HAR70+ and HARTH. This versatility is critical in IoMT-based HAR applications, which often involve heterogeneous sensor data collected from distributed devices.

Cruciani et al. [20] reported an accuracy of 91.98% on UCI-HAR using spatial feature extraction methods. However, these approaches struggle to fully capture the temporal complexity of human activity data. In contrast, the proposed framework’s accuracy of 95.6% ± 0.3 demonstrates the advantages of hybrid models that effectively integrate spatial and temporal features. This capability is crucial for accurate classification in privacy-sensitive federated environments.

Logacjov et al. [21] employed self-supervised learning and achieved 93.8% on HAR70+ and 94.6% on HARTH, benefiting scenarios with limited labeled data. The proposed model surpasses these results with 95.2% ± 0.5 on HAR70+ and 94.9% ± 0.6 on HARTH, reflecting stronger generalizability and robustness. This performance advantage is critical in federated learning scenarios where labeled data may be scarce, yet high accuracy is essential for effective real-time activity recognition.

Sahoo et al. [22] achieved an accuracy of 88.89% on HARTH by optimizing feature selection. However, their approach was limited in achieving higher classification accuracy. In comparison, the proposed framework achieves 94.9% ± 0.6, benefiting from a more integrated approach to feature extraction and classification. By combining federated learning with the hybrid LSTM-GRU model, the framework ensures enhanced classification accuracy while preserving privacy, making it well-suited for IoMT applications.

## 6. Discussion

Despite considerable progress in human activity recognition, most existing research has focused on centralized data processing models that rely on deep learning algorithms to analyze sensor data from mobile and wearable devices. While these centralized approaches can be effective in controlled environments, they face significant challenges in real-world applications, especially in terms of privacy and adaptability for decentralized IoMT environments. The proposed study addresses this gap by introducing a federated learning framework paired with a hybrid LSTM-GRU model, specifically crafted for decentralized human activity recognition tasks in IoMT settings.

This study demonstrates that federated learning, when combined with a hybrid LSTM-GRU model, can maintain high classification accuracy for human activity recognition while also addressing critical privacy issues linked to centralized data processing. By utilizing federated learning, the proposed approach ensures that sensitive data remain on the local devices, mitigating privacy risks without sacrificing model quality. The dual-feature extraction mechanism, which integrates convolutional layers for capturing local data patterns with the LSTM-GRU model for detecting complex temporal dependencies, proves effective in handling the nuanced nature of real-world activity data. This combination enables the model to accurately capture both short-term and long-term dependencies in activity patterns, providing a balanced and holistic view of human activities across various real-life contexts.

A key consideration in our research was the selection of appropriate datasets to validate our approach. While it is true that data collected directly from medical devices is often sensitive and subject to stringent privacy regulations, there exist medical datasets that are openly available for research purposes under specific conditions. These datasets enable researchers to develop and test models in healthcare-oriented settings while ensuring the protection of sensitive information. Although our current study primarily utilized the UCI-HAR, HARTH, and HAR7+ datasets, which are collected from healthy individuals using consumer-grade devices, our federated learning framework is inherently compatible with medical-grade data sources. This adaptability allows for future integration of specialized medical datasets, thereby extending the applicability of our model to more diverse and clinically relevant scenarios.

Furthermore, the datasets employed in our research are highly consistent with the daily activities of individuals, aligning well with the objectives of IoMT-enhanced HAR. These activities mirror real-world scenarios, providing a robust foundation for developing models that can be effectively deployed in various IoMT contexts, including health monitoring and daily living assistance. In the realm of IoMT, human activity data are often gathered through mobile devices such as smartphones and non-medical sensors rather than exclusively relying on medical-grade equipment. This approach is prevalent in most similar studies and reflects the practical deployment of IoMT systems where mobile and consumer-grade sensors play a significant role in data collection. By leveraging these widely used data sources, our federated learning framework and hybrid LSTM-GRU model remain relevant and applicable to the types of data commonly encountered in IoMT environments.

Moreover, our Discussion Section has been revised to emphasize the generalizability and scalability of our proposed framework. The author elaborates on how the architecture can seamlessly integrate diverse data sources, whether they originate from medical-grade devices or consumer-grade sensors, ensuring both privacy preservation and high recognition accuracy. This flexibility is crucial for adapting to the dynamic and heterogeneous nature of IoMT ecosystems. Additionally, I highlight the potential for future research to incorporate more specialized medical datasets, which would further validate and enhance the applicability of our approach in real-world medical settings. By addressing these aspects, I demonstrate that our framework is not only grounded in robust and widely accepted datasets but is also poised to evolve in line with the specific demands of healthcare-oriented IoMT applications.

Our findings confirm the capability of the LSTM-GRU model to outperform single-recurrent models by effectively capturing sequential dependencies and dynamic patterns in sensor data. This feature makes the hybrid model particularly well-suited for human activity recognition tasks that require continuous monitoring and real-time data processing, as seen in IoMT applications. The federated learning approach further enhances the model’s scalability, allowing it to be deployed across diverse IoMT devices without compromising performance or privacy.

Despite these promising results, our study has certain limitations. One significant challenge is the variation in data quality across different sensors and devices, which can affect model performance in heterogeneous IoMT networks. Additionally, the current framework utilizes a static federated learning protocol that may not fully optimize data exchange across devices with different computational capabilities. Future research should explore adaptive federated learning protocols that adjust dynamically to device-specific constraints, thereby enhancing the framework’s scalability and efficiency across various IoMT environments.

Our work also paves the way for further research, particularly in enhancing privacy and robustness in decentralized human activity recognition systems. Integrating additional privacy-preserving techniques, such as differential privacy or homomorphic encryption, could further secure data exchanges within federated learning frameworks. Investigating advanced deep learning architectures and regularization techniques may also improve model resilience to noisy data, making it more adaptable to real-world conditions. Expanding this framework to include adaptive feedback for real-time activity guidance and anomaly detection could broaden its practical applicability.

The practical implications of this study are significant for advancing IoMT-driven human activity recognition systems. By leveraging federated learning, the proposed framework ensures privacy-preserving data processing, which is essential for applications in sensitive domains such as healthcare and smart homes. Its scalability and adaptability to decentralized, resource-constrained environments make it suitable for real-world deployments. Additionally, the integration of Weighted Federated Averaging effectively addresses class imbalance, enhancing the robustness and fairness of activity detection in practical scenarios

Overall, our study offers a robust and scalable approach to human activity recognition in IoMT settings, merging the benefits of federated learning and hybrid LSTM-GRU networks. The strong performance of our framework underscores its potential as a privacy-preserving, flexible solution for decentralized human activity recognition applications. Future research should focus on refining federated learning processes, incorporating enhanced privacy-preserving techniques, and validating the framework across a broader range of IoMT devices and datasets. This work represents a significant step forward, contributing toward more secure and intelligent human activity recognition systems tailored for real-world, privacy-conscious environments.

## 7. Conclusions

This study presents a federated learning framework combined with a hybrid LSTM-GRU model for human activity recognition within IoMT-enhanced environments. Our approach addresses the growing demand for high-accuracy human activity recognition systems that simultaneously ensure data privacy by decentralizing the training process. By leveraging federated learning, the proposed framework enables local model updates on individual devices, thereby minimizing the need for centralized data aggregation and reducing privacy risks. The integration of convolutional layers with a hybrid LSTM-GRU structure enhances feature extraction capabilities, allowing the model to capture both local patterns and complex temporal dependencies in activity data. Experimental results across multiple datasets, including HAR70+, UCI-HAR, and HARTH, demonstrate that our model achieves superior performance compared to existing methods, both in classification accuracy and privacy preservation. These results affirm the effectiveness of combining federated learning with hybrid recurrent networks to create a scalable and reliable human activity recognition solution for real-world IoMT applications.

While the proposed framework achieves promising results, there are areas for further enhancement. Future work could explore adaptive federated learning protocols that dynamically adjust to device-specific constraints, thereby improving efficiency and scalability across heterogeneous IoMT devices with varying computational resources. Additionally, integrating advanced privacy-preserving techniques, such as differential privacy or homomorphic encryption, could further safeguard data during model updates and exchanges. To enhance model robustness in diverse environments, future studies should consider more sophisticated deep learning architectures or regularization techniques to increase resilience to noise and variability in sensor data. Testing the framework on larger and more varied datasets will also provide a stronger basis for evaluating its generalizability and effectiveness in handling a wider range of human activities. Finally, I aim to incorporate medical-grade datasets to further validate and enhance the applicability of our federated learning framework within healthcare-oriented IoMT environments. Collaborating with healthcare providers will be essential to access sensitive medical data under strict privacy protocols, allowing us to develop more specialized HAR models tailored to clinical settings. Additionally, the author plans to integrate a broader range of IoMT devices, including advanced medical sensors, to capture a more comprehensive set of human activities relevant to patient monitoring and health management. Exploring the impact of various privacy-preserving techniques on model performance in medical contexts will also be a key focus, ensuring compliance with healthcare data regulations while maintaining high recognition accuracy. I intend to investigate the scalability of our framework in larger and more diverse IoMT deployments, addressing real-world challenges such as heterogeneous data sources and varying device capabilities. By addressing these areas, the author seeks to advance the robustness and practical applicability of the proposed HAR solutions in the evolving landscape of the Internet of Medical Things.

## Figures and Tables

**Table 1:** Summary of related works.

Reference	Approach	Methodology	Dataset	Overall Accuracy for Related Datasets
Heba, N. and Sreeraman, R. [16]	Wavelet transform with optimal mother wavelet selection for precise HAR	Wavelet packet transform combined with energy-to-Shannon-entropy ratio, classification with DT and SVM	MHEALTH, WISDM Activity Prediction, HARTH, HARsense, DaLiAc, PAMAP2, REALDISP, HAR70+	HAR70+: 84.64% Harth: 75.73%
Chen, K. et al. [17]	Lightweight machine unlearning for federated learning human activity recognition.	Utilizes a third-party dataset unrelated to model training to refine the FL-HAR model by selectively removing specific portions of a client’s training data. KL divergence is employed as the loss function, with membership inference evaluation used.	HAR70+, HARTH, MNIST	HAR70+: 97% HARTH: 91%
Gehlhar, Till et al. [18]	Secure MPC-based framework for robust federated learning against combined attacks	Multi-party computation (MPC) protocols to secure federated learning against privacy and poisoning attacks	UCI-HAR	UCI-HAR: 97%
Koşar, E. and Barshan, B. [19]	Hybrid CNN-LSTM architecture for feature extraction from motion sensor data	Parallel CNN and LSTM branches for spatial and temporal feature extraction, concatenation for classification	UCI HAR, daily and sports activities (DSA) dataset	UCI-HAR: 95.66%
Cruciani, F. et al. [20]	CNN-based feature learning for automated activity recognition	Pre-trained CNN feature extractor assessed on multiple topologies and parameters	UCI-HAR, extrasensory, DCASE 2017	UCI-HAR: 91.98%
Logacjov, A et al. [21]	Self-supervised learning for HAR using large-scale unlabeled accelerometer data	Transformer encoder network for pre-training on masked accelerometer data, SSL for downstream HAR tasks	HUNT4 (self-supervised pre-training), HARTH, HAR70+, PAMAP2, Opportunity, RealWorld	HAR70+: 93.8% HARTH: 94.6%
Sahoo, KK et al. [22]	Wrapper-based deep feature optimization for efficient and accurate HAR	Transfer learning with CNN-based feature extraction, followed by wrapper method for feature selection	HARTH, KU-HAR, HuGaDB	HARTH: 88.89%

**Table 2:** Dataset summary.

Dataset Name	Number of Labels	Number of Subjects	Sensor Type	Type of Annotation
HARTH	12	22	Axivity AX3	Video and Human Experts
HAR70+	8	18	Axivity AX3	Video
UCI-HAR	6	30	Smartphone Sensors	Manual Labeling

**Table 3:** Centralized vs. federated learning experiment results.

Dataset	Learning Type	Accuracy (Mean ± SD)	F1-Score (Mean ± SD)	Precision (Mean ± SD)	Recall (Mean ± SD)
HARTH	Centralized	92.7% ± 0.4	0.91 ± 0.02	0.91 ± 0.02	0.90 ± 0.02
	Federated	94.9% ± 0.6	0.93 ± 0.02	0.92 ± 0.03	0.91 ± 0.03
HAR70+	Centralized	90.8% ± 0.6	0.89 ± 0.03	0.88 ± 0.04	0.87 ± 0.03
	Federated	95.2% ± 0.5	0.91 ± 0.02	0.90 ± 0.03	0.89 ± 0.03
UCI-HAR	Centralized	94.8% ± 0.3	0.93 ± 0.01	0.92 ± 0.02	0.92 ± 0.02
	Federated	95.6% ± 0.3	0.94 ± 0.01	0.93 ± 0.02	0.93 ± 0.02

**Table 4:** Class imbalance results.

Dataset	Class Imbalance Handling	Accuracy (Mean ± SD)	F1-Score (Mean ± SD)
HARTH	Before	88.5% ± 0.8	0.83 ± 0.03
	After	94.9% ± 0.6	0.93 ± 0.02
HAR70+	Before	86.2% ± 0.9	0.81 ± 0.04
	After	95.2% ± 0.5	0.91 ± 0.02
UCI-HAR	Before	89.0% ± 0.7	0.85 ± 0.03
	After	95.6% ± 0.3	0.94 ± 0.01

**Table 5:** Class-wise accuracy and F1-score for the HARTH dataset.

Activity	Accuracy (Before)	Accuracy (After)	F1-Score (Before)	F1-Score (After)
Walking	92.5%	96.8%	0.88	0.95
Running	70.3%	85.0%	0.65	0.82
Cycling	72.1%	86.2%	0.67	0.83
Sitting	88.0%	92.5%	0.84	0.91
Standing	89.3%	94.1%	0.86	0.93
Others (6 activities)	84.0%	90.2%	0.78	0.88

**Table 6:** Cross-dataset generalization experiment results.

Training Dataset	Test Dataset	Accuracy (Before)	Accuracy (After FedAvg)
HARTH	HAR70+	82.3% ± 1.1	90.8% ± 0.8
HAR70+	HARTH	83.1% ± 1.0	91.5% ± 0.7
UCI-HAR	HAR70+	86.5% ± 0.8	95.2% ± 0.5
HAR70+	UCI-HAR	87.6% ± 0.7	98.7% ± 0.3

**Table 7:** Class-wise accuracy.

Activity	Accuracy (Before)	Accuracy (After FedAvg)	F1-Score (Before)	F1-Score (After FedAvg)
Walking	92.5% ± 0.5	97.0% ± 0.4	0.89 ± 0.02	0.96 ± 0.01
Running	71.0% ± 1.2	86.2% ± 0.9	0.66 ± 0.04	0.84 ± 0.03
Cycling	73.0% ± 1.1	87.0% ± 0.8	0.68 ± 0.04	0.84 ± 0.03
Sitting	88.2% ± 0.7	93.0% ± 0.5	0.85 ± 0.03	0.92 ± 0.02
Standing	89.5% ± 0.6	94.3% ± 0.4	0.87 ± 0.03	0.94 ± 0.02
Others (6)	84.5% ± 0.8	91.0% ± 0.7	0.79 ± 0.04	0.89 ± 0.03

**Table 8:** Stability metrics.

Communication Rounds	HARTH Accuracy	HARTH F1-Score	HAR70+ Accuracy	HAR70+ F1-Score	UCI-HAR Accuracy	UCI-HAR F1-Score
Mean	91.4% ± 0.7	0.89 ± 0.03	89.2% ± 1.1	0.86 ± 0.04	92.0% ± 0.4	0.89 ± 0.03
Min	87.1%	0.82	85.0%	0.78	88.5%	0.83
Max	91.7%	0.89	89.5%	0.87	92.0%	0.89
IQR	3.6%	0.05	4.5%	0.07	2.5%	0.06

**Table 9:** Evaluation results of validation accuracy for HAR70+, HARTH, and UCI-HAR datasets for the proposed work and previous work.

Work	Approach	Dataset	Accuracy (Mean ± SD)
Heba, N., et al. [16]	Wavelet Transform for HAR	HAR70+	84.64% ± 1.2
		HARTH	75.73% ± 1.4
Gehlhar, T., et al. [18]	Secure MPC-based Federated Learning	UCI-HAR	97.0% ± 0.5
Koşar, E., et al. [19]	Hybrid CNN-LSTM	UCI-HAR	95.66% ± 0.6
Cruciani, F., et al. [20]	CNN Feature Learning	UCI-HAR	91.98% ± 0.7
Logacjov, A., et al. [21]	Self-Supervised Learning for HAR	HAR70+	93.8% ± 0.6
		HARTH	94.6% ± 0.5
**Proposed Work**	**Federated LSTM-GRU**	**HAR70+**	**95.2% ± 0.5**
		**UCI-HAR**	**95.6% ± 0.3**
		**HARTH**	**94.9% ± 0.6**

## Data Availability

The data presented in this study are openly available in [HARTH: a human activity recognition dataset for machine learning] [https://doi.org/10.3390/s21237853] [27].
